# Full-Genome Sequence of *Escherichia coli* K-15KW01, a Uropathogenic *E. coli* B2 Sequence Type 127 Isolate Harboring a Chromosomally Carried *bla*_CTX-M-15_ Gene

**DOI:** 10.1128/genomeA.00927-16

**Published:** 2016-09-01

**Authors:** Katrin Zurfluh, Taurai Tasara, Roger Stephan

**Affiliations:** Institute for Food Safety and Hygiene, Vetsuisse Faculty, University of Zurich, Zürich, Switzerland

## Abstract

We present here the full-genome sequence of *Escherichia coli* K-15KW01, an extended-spectrum-β-lactamase-producing uropathogenic strain. Assembly and annotation of the draft genome resulted in a 5,154,641-bp chromosome and revealed a chromosomally contained *bla*_CTX-M-15_ gene embedded at the right-hand extremity of an IS*Ecp1* element in a plasmid-like structure (36,907 bp).

## GENOME ANNOUNCEMENT

The production of extended-spectrum β-lactamases (ESBLs) is one of the most important mechanisms of antibacterial resistance in *Enterobacteriaceae*. Most ESBLs can be divided into 4 groups: TEM, SHV, OXA, and CTX-M types (1). Currently, CTX-Ms are the most prevalent type of ESBLs described (2, 3). The last decade has seen a high degree of diversification of these enzymes and an explosive global spread driven primarily by their carriage on resistance plasmids and by the spread of extraintestinal pathogenic *Escherichia coli* clones (4). Meanwhile, it has become widely recognized that the dissemination of ESBL-producing bacteria is an issue that is no longer restricted to the medical health care system. In the last few years, there has been an increase in the detection of ESBL-producing strains in the general community (5, 6).

In a recent study, we isolated an ESBL-producing *E. coli* isolate (B2 sequence type 127 [ST127]) harboring a *bla*_CTX-M-15_ gene from a healthy asymptomatic carrier shedding the same strain for 5 months in fecal samples (7). Despite repeated attempts, the *bla*_CTX-M-15_ gene from this isolate could not be transferred by conjugation. Southern blot hybridization provided evidence for a chromosomal location of *bla*_CTX-M-15_ (data not shown). Therefore, genomic DNA was isolated from K-15KW01 and subjected to sequencing using Pacific Biosciences single-molecule real-time (SMRT) technology at the ChunLab at Seoul National University, South Korea. The K-15KW01 genome was assembled *de novo* using the SMRT Analysis 2.3.0 software to a single chromosome of 5,154,641 bp in size, with a G+C content of 50.4%. Genome annotation was conducted through the NCBI Prokaryotic Genome Annotation Pipeline (PGAP) (http://www.ncbi.nlm.nih.gov/genome/annotation_prok/) (8).

By full-genome sequencing, the chromosomal location of the *bla*_CTX-M-15_ gene could be proven. The *bla*_CTX-M-15_ gene was embedded at the right-hand extremity of an IS*Ecp1* element in a plasmid-like structure (36,907 bp) located between a putative gene encoding for an oxalyl-coenzyme A (CoA) decarboxylase domain (positions 978270 to 978743) and a formyl-CoA-oxalate CoA-transferase (gene *frc*) (positions 1015704 to 1016954). Moreover, on this plasmid-like structure, a tunicamycin resistance determinant (*tmrB*), an aminoglycoside N(3′)-acetyl transferase III (*aacC2*), the cr variant of *aac(6′)-Ib* encoding an aminoglycoside acetyltransferase conferring resistance to fluoroquinolones, the β-lactamase gene *bla*_OXA-1_, and the chloramphenicol acetyltransferase *catB3* were carried.

*E. coli* B2:ST127 has recently been described as an emerging clone with extremely high uropathogenic potential (9) and has been associated with community-acquired urinary tract infections (CAUTI) in Britain (10). The detection of uropathogenic *E. coli* (UPEC) B2:ST127 isolates harboring chromosomally carried *bla*_CTX-M-15_ in a healthy person is a matter of concern, since UPEC isolates spread easily via person-to-person contact or fecal-oral transmission (11).

### Accession number(s).

Sequence and annotation data of the genome of *E. coli* strain K-15KW01 were deposited in the GenBank database with the accession no. CP016358.

## References

[1] Bush K, Jacoby GA 2010 Updated functional classification of beta-lactamases. Antimicrob Agents Chemother 54:969–976. doi:10.1128/AAC.01009-09. 19995920 PMC2825993

[2] Cantón R, González-Alba JM, Galán JC 2012 CTX-M enzymes: origin and diffusion. Front Microbiol 3:110. doi:10.3389/fmicb.2012.00110. 22485109 PMC3316993

[3] Naseer U, Sundsfjord A 2011 The CTX-M conundrum: dissemination of plasmids and *Escherichia coli* clones. Microb Drug Resist 17:83–97. doi:10.1089/mdr.2010.0132. 21281129

[4] Cantón R, Coque TM 2006 The CTX-M beta-lactamase pandemic. Curr Opin Microbiol 9:466–475. doi:10.1016/j.mib.2006.08.011. 16942899

[5] Luvsansharav UO, Hirai I, Niki M, Nakata A, Yoshinaga A, Moriyama T, Yamamoto Y 2011 Prevalence of fecal carriage of extended-spectrum β-lactamase-producing *Enterobacteriaceae* among healthy adult people in Japan. J Infect Chemother 17:722–725. doi:10.1007/s10156-011-0225-2. 21359543

[6] Geser N, Stephan R, Korczak BM, Beutin L, Hächler H 2012 Molecular identification of extended-spectrum-β-lactamase genes from *Enterobacteriaceae* isolated from healthy human carriers in Switzerland. Antimicrob Agents Chemother 56:1609–1612. doi:10.1128/AAC.05539-11. 22155836 PMC3294945

[7] Zurfluh K, Hächler H, Stephan R, Nüesch-Inderbinen M. Long-term shedding of CTX-M-15 producing. *E. coli* B2:ST127 by a healthy asymptomatic carrier. Int J Antimicrob Agents, in press. 10.1016/j.ijantimicag.2016.08.004 27637466

[8] Angiuoli SV, Gussman A, Klimke W, Cochrane G, Field D, Garrity G, Kodira CD, Kyrpides N, Madupu R, Markowitz V, Tatusova T, Thomson N, White O 2008 Toward an online repository of Standard Operating Procedures (SOPs) for (meta)genomic annotation. Omics 12:137–141. doi:10.1089/omi.2008.0017. 18416670 PMC3196215

[9] Alghoribi MF, Gibreel TM, Dodgson AR, Beatson SA, Upton M 2014 *Galleria mellonella* infection model demonstrates high lethality of ST69 and ST127 uropathogenic *E. coli*. PLoS One 9:e101547. doi:10.1371/journal.pone.0101547. 25061819 PMC4111486

[10] Gibreel TM, Dodgson AR, Cheesbrough J, Fox AJ, Bolton FJ, Upton M 2012 Population structure, virulence potential and antibiotic susceptibility of uropathogenic *Escherichia coli* from Northwest England. J Antimicrob Chemother 67:346–356. doi:10.1093/jac/dkr451. 22028202

[11] Foxman B 2010 The epidemiology of urinary tract infection. Nat Rev Urol 7:653–660. doi:10.1038/nrurol.2010.190. 21139641

